# Traumatic Tetanus

**Published:** 1869-08-15

**Authors:** 


***Traumatic Tetanus.***

Dr. Buchon (of Quingey) reports to the *Gazette des Hopitaux*, of May 29, a case of tetanus, resulting from extensive burns upon the lower extremities, occasioned by falling into a vessel of boiling acidulated water. The patient being a factory operative, aged thirty-eight years, immediately after the accident, had plunged into a river. The tetanic symptoms, supervening upon the eleventh night, were ushered in by recurrent lancinating pains in the left heel (that of the burned leg), extending into the leg and foot, occasioning cramps and separation of the toes (in the language of the patient "like a fan or a pigeon's wing"), closing of the jaws which, even during the intervals of the paroxysms, could not be freely opened. The determining cause of this complication appears to have been cold resulting from the diminished temperature of the room, occasioned by leaving a door open during the fifth night. The patient was first treated with laudanum, the result of which, after two days, was an exaggeration of all his symptoms already existing, with the addition of great gastric irritation, for which seidlitz was prescribed—opium having thus far failed, as chloroform on two former occasions. Dr. Buchon next administered bromide of potassium, four grammes (about a drachm) during the day. This was followed by slight amelioration of the paroxysms and diminution of the sweat; but three days afterwards the lancinating pains in the heel, with the cramps, returned with all their former intensity. An addition of one gramme (15 grs.), to the dose, of bromide, resulted in a new amelioration. Suspecting the establishment of tolerance, and to avoid being forced by the intensity of the paroxysms to increase the dose, in order to control them, Dr. B. added, every two days, one gramme (15 grs.) of bromide to the quantity already administered (daily). This gradual increase was continued up to eight grammes (about two drachms) daily, and resulted in gradual diminution, and finally in complete cessation of the disease.

Dr. B. remarks further that the paroxysms occurred sometimes spontaneously, even under the influence of five grammes of the salt daily, although they were then most frequently reflex, the slightest touch upon the great toe of the left side being sufficient to induce a recurrence.

There was likewise in this patient, during the latter part of the treatment, a brownish (bistre) discoloration of the skin (he asks, was it an effect of the bromide?)

It was moreover demonstrated, in this case, to be necessary to administer the salt in divided doses, at short intervals, rather than in larger quantities, after longer periods of time, so that the patient may be kept continually under its influence until the final extinction of the disease.

